# Unveiling the common loci for six body measurement traits in Chinese Wenshan cattle

**DOI:** 10.3389/fgene.2023.1318679

**Published:** 2023-11-24

**Authors:** Honghui Ren, Xiaoming He, Ying Lu, Dan Yue, Xingneng Liu, Dongwang Wu, Junhong Zhu, Zhendong Gao, Dongmei Xi, Weidong Deng

**Affiliations:** ^1^ Yunnan Provincial Key Laboratory of Animal Nutrition and Feed, Faculty of Animal Science and Technology, Yunnan Agricultural University, Kunming, China; ^2^ Sichuan Ganzi Tibetan Autonomous Prefecture Institute of Animal Husbandry Science, Kangding, China; ^3^ Institute of Animal Husbandry, Yunnan Vocational College of Agriculture, Kunming, China; ^4^ Faculty of Animal Science and Technology, Yuxi Agricultural Vocational and Technical College, Yuxi, China

**Keywords:** Wenshan cattle, body measurement traits, SNPs, genome-wide association study, genetic selection

## Abstract

**Introduction:** Body measurement traits are integral in cattle production, serving as pivotal criteria for breeding selection. Wenshan cattle, a local breed in China’s Yunnan province, exhibit remarkable genetic diversity. However, the molecular mechanisms regulating body measurement traits in Wenshan cattle remain unexplored.

**Methods:** In this study, we performed a genome-wide association method to identify genetic architecture for body height body length hip height back height (BAH), waist height and ischial tuberosity height using the Bovine 50 K single nucleotide polymorphism Array in 1060 Wenshan cattles.

**Results:** This analysis reveals 8 significant SNPs identified through the mixed linear model (MLM), with 6 SNPs are associated with multiple traits and 4 SNPs are associated with all 6 traits. Furthermore, we pinpoint 21 candidate genes located in proximity to or within these significant SNPs. Among them, *Scarb1*, *acetoacetyl-CoA synthetase* and *HIVEP3* were implicated in bone formation and rarely encountered in livestock body measurement traits, emerge as potential candidate genes regulating body measurement traits in Wenshan cattle.

**Discussion:** This investigation provides valuable insights into the genetic mechanisms underpinning body measurement traits in this unique cattle breed, paving the way for further research in this domain.

## 1 Intruduction

The nutritional value of beef, characterized by its high-quality animal protein content, underscores its significance in the meat market (Wu G, 2020). With an escalating demand for beef due to the increased consumption, understanding and enhancing the economic traits associated with beef cattle have become paramount (MacDonald, 2003). Body measurement traits, such as body length (BL), body height (BH) and back height (BAH), are widely used as important indicators for selection criterion to improve the production of the beef cattle (Gritsenko et al., 2023). According to previous study, these body measurement traits have demonstrated correlations with various other economically significant characteristics, such as reproductive traits (Bessa et al., 2021; Shin et al., 2021), longevity (Forabosco et al., 2004; Setati et al., 2004), feed efficiency (Benfica et al., 2020), carcass traits (Munim, et al., 2013) and growth traits (Naserkheil et al., 2020). Furthermore, body measurement traits can also be used to predict cattle’s health status (Schmidtmann et al., 2023). In the past few decades, the improvement of beef cattle body measurements has relied on conventional breeding techniques. Body measurement traits, as a kind of quantitative traits which are controlled by a few major genes and numerous minor genes, are also influenced by various environmental variables, such as diet, prenatal conditions and living environment (Larson et al., 2023; Dawson et al., 2022). Thus, the efficacy of traditional methods is constrained (Ring et al., 2019). Fortunately, due to the reduction in sequencing costs, the completion of the cattle genome sequence, and the continuous improvement of the genetic evaluation methods, molecular marker-assisted breeding has become an effective and reliable way to improve beef cattle’s body measurement traits (Naserkheil et al., 2020).

Wenshan cattle, colloquially referred to as Wenshan-humped yellow cattle, represent a distinctive beef cattle breed primarily found in the Wenshan Zhuang and Miao Autonomous Prefecture of Yunnan Province in China. Wenshan cattle has excellent features like adaptation to rough feed, stress resistance capacity, early mature (China National Commission of Animal Genetic Resources, 2011), tolerance to hot and humid environments (Liu et al., 2022; Yan et al., 2022), superior meat quality, and a wealth of genetic diversity (Nie et al., 1999; Chen et al., 2020), and has important value of research. Chen, Zhi et al. (2019)showed that the contents of fat, myristic acid, palmitic acid and palmitoleic acid in the longissimus muscle of 1-year old Wenshan bull were higher than those of Simmental cattle. However, akin to other beef cattle breeds, Wenshan cattle have their inherent limitations. As to 18-month old cattle, the average body weight of Wenshan cattle was significantly lower than Simmental cattle (*p* = 0.0004) (Li et al., 2019). The growth performance and carcass traits of Wenshan cattle were also lower than those of Yunling cattle and Simmental cattle, respectively (Fan et al., 2020). At 12 months, Wenshan cattle display notably smaller body measurements, including withers height, body slanting length, chest circumference, and hip and rump length, compared to Yunling cattle and Simmental cattle (*p* < 0.05) (Meng et al., 2020). These deficiencies, such as smaller size, long growth cycle and lower growth rate, along with diminished meat production performance and higher feeding costs in comparison to widely used breeds, which restrict its further development. Therefore, improvement in the growth traits is the key to the development of Wenshan cattle industry.

With the rapid development of single nucleotide polymorphism (SNP) arrays and the reduction of the genotyping costs, genome-wide association study (GWAS) has been widely used to identify genomic regions that control economic traits in domestic animals (Zhang et al., 2019; Song et al., 2023). GWAS employs thousands of SNP markers to efficiently associate economic traits in beef cattle, such as body measurement traits, and many candidate genes has been identified in recent years (van den Berg et al., 2022; Niu et al., 2021; Bouwman et al., 2018). In a recent study, 463 Wagyu beef cattles were genotyped by 770K SNP array to identify candidate genes for body measurement traits, and 18, 5 and 1 SNPs associated with hip height, body height and body length were detected respectively through GWAS (An et al., 2019). Vanvanhossou et al. (2020) detected that PIK3R6 and PIK3R1 had direct functional associations with height and body size for 4 Beninese indigenous cattle breeds. Terakado, et al. (2018) identified that the gene TMEM68 had associated with yearling height in Nelore cattle using GWAS based on the Illumina BovineHD BeadChip. Recently, An et al. (2020) conducted GWAS on 1,217 Chinese Simmental beef cattle to study the changes of body size traits such as heart size (HS), abdominal size (AS), body height (BH), body length (BL), hip height (HH) and cannon bone size (CS) at three different growth stages (6, 12 and 18 months). In the above research, Illumina Bovine HD 770 K BeadChip was used through GWAS, and revealed 58 significant SNPs compatible with 21 genes correlated to body size in Simmental beef cattle. Due to the difficulty in collecting phenotype data and the high cost of large population sequencing, studies on the molecular mechanisms of body size traits in beef cattle remain limited. However, GWAS analyses face a significant challenge in controlling false positives and false negatives that may arise due to population structure and kinship. To solve this problem, mixed linear models (MLMs) were frequently used, which incorporate covariates for population structure and principal component analysis (PCA). Linkage disequilibrium (LD) serves as the foundation of GWAS, which provided insight in identifying genomic regions to improve economically important traits (McKay et al., 2007; Porto-Neto et al., 2014). LD is mainly determined by the physical distance between markers, and may also be influenced by several other demographic and evolutionary factors including genetic bottleneck, population stratification, selection, inbreeding, genetic drift, effective population size, migration, mutation, and recombination rate (Reich et al., 2011; Karimi et al., 2015; Singh et al., 2021). Consequently, GWAS is suitable for studying body measurement traits.

In this study, the GWAS was conducted using the Bovine 50K SNP Array to identify significant SNPs associated with six body measurement traits of 1060 Wenshan cattles, including body height (BH), body length (BL), hip height (HH), back height (BAH), waist height (WH) and ischial tuberosity height (ITH). To enhance the accuracy of our analysis and control for population structure, we employed the Mixed Linear Model (MLM) and used population structure as covariates. The primary objective of this study was to map significant SNPs, with a particular focus on common SNPs, and to identify candidate genes that play a role in body measurement-related traits. Besides, Mixed Linear Model (MLM) was used for GWAS analysis, and population structure was used as covariates. The objective of this study was to map significantly associated SNPs, especially common SNPs, and to identify candidate genes involved in body measurement related traits. This study represents the first comprehensive genome-wide investigation of body measurement-related traits in Wenshan cattle, with the aim of identifying candidate genes and potential markers. The findings of this research provide valuable insights and pave the way for further exploration of the genetic mechanisms underlying body measurement traits in Wenshan cattle. This study offers valuable insights for the further investigation of potential genetic mechanism of body measurement traits in Wenshan cattle.

## 2 Materials and methods

### 2.1 Ethics statement

All of the animals employed in this research were handled following the guidelines established by the Ministry of Agriculture and Rural Affairs of China for use of experimental animals. The ethics committee of Yunnan Agricultural University (YNAU, Kunming, China) approved of the entire research.

### 2.2 Animals, phenotypic collection and statistical analysis

According to the date of birth, 1,060 Wenshan cattles (900 females and 160 males) aged around 12-monthold (±15 days) were collected from Yunnan Guduo Agriculture and Animal Husbandry Co., Ltd. in Yunnan Province, China. These cattles were reared under the homologous management conditions and similar environments. In this study, 6 phenotypic traits including BH, BL, WH, BAH, ITH and HH were measured simultaneously for each individual by the method as in Ref. (An et al., 2020). Ear tissue samples were collected from the up 1,060 Wenshan cattles, and were stored at −80°C.

SPSS25 software was used to make descriptive statistics, including the number, minimum, maximum, mean, standard deviation, and coefficient of variation for our 6 body measurement traits in Wenshan cattle. Phenotypic correlations among the body measurement traits were calculated within the R statistical environment and used to determine whether they reflected the relationships between the GWAS results.

### 2.3 Genotyping and quality control

Ear samples of 1,060 Wenshan cattle were used to extract the DNA using the Tissues Genomic DNA (Omega Bio-Tek, Norcross, GA, United States) kit according to the manufacturer’s instructions. After that, the DNA was quantified and genotyped using the Bovine 50K SNP Array and the PLINK v1.90 Software was used for quality control (Purcell et al., 2007). Briefly, The raw genotypic data were controlled according to call rates >0.95 and markers with call rates >0.95, minor allele frequency (MAF) > 0.01, and Hardy–Weinberg (HWE) *p* > 10^–6^ were retained. All markers located on sex chromosomes or in unmapped regions were excluded. Missing genotypes were imputed using the Beagle software (Browning and Browning, 2009). After that, 46,284 high-quality SNPs were used for subsequent analyses.

### 2.4 Population structure

To investigate population structure of Wenshan cattle, population stratification was detected using eigenvalues and eigenvectors calculated by GCTA software (Li et al., 2016) based on principal component analysis (PCA). The SNPs density distributions plot was drawn using the R package “CMplot” (Yin, L. et al., 2021).

### 2.5 Association analysis

To test for normality, the Shapiro test was performed on our phenotypic data of 6 body measurement traits. Since the original data are not normally distributed, a log transformation was performed to normalize it. The normalized data on the 6 body measurement traits were then subjected to a one-way analysis of variance (ANOVA), followed by *post hoc* Fisher’s least significant difference (LSD) test using the Agricolae package (Steel and Torrie, 1997) in R 4.2 (https://www.R-project.org/). The log-transformed phenotypic data were eventually used in the GWAS analysis. The single variable mixed linear model (MLM) of GEMMA software (Zhou and Stephens, 2012) was employed for association analysis between body measurement traits and filtered SNP markers (n = 46,284). The first three principal components of principal component analysis were used as covariables to conduct association analysis. The univariate linear mixing model is as follows:

y=γPca+Xβ+Zα+Wμ+e

Where y is the phenotypic value vector; γ is the regression coefficient; PCA is a covariate vector; β is the fixed effect vector of birth year, birth month and sex; α is SNP effect value vector; μ is the residual polygenic effect value vector, which follows the [a∼MVN(0, A $\sigma_{a}^{2}$ )] distribution, and A represents the molecular affinity matrix. e is the residual effect value vector, following the [e ∼ MVN(0, I $\sigma_{e}^{2}$ )] distribution, I represents the unit vector; X, Z and W are the correlation matrices of β, α and μ, respectively.

Using the Bonferroni correction, the genome-wide significant thresholds and suggestion threshold were set as *p* < 0.05/N, *p* < 1/N, respectively, where N was the number of SNPs tested in the analyses. Genome-wide significant and suggestive levels were set as 0.05⁄46,284 = 1.08 × 10^−6^ and 1⁄46,284 = 2.16 × 10^−5^, respectively. Finally, the manhattan and Quantile-Quantile (Q-Q) plots of GWAS were drawn using the R package “qqman”.

### 2.6 Candidate gene selection and functional annotations

Candidate genes were selected within 0.25 Mb upstream or downstream of the significant SNPs based on LD value for Wenshan cattle population. The major genome browser, Ensembl (https://uswest.ensembl.org/index.html), was used to annotate the significant SNPs identified for the 6 body measurement traits in Wenshan cattle. Candidate genes were then selected according to their biological function.

## 3 Results

### 3.1 Phenotype data statistics

The statistical information on the six body measurement traits is shown in Table 1. The mean values for BL, BH, WH, ITH, BAH and HH were 101.58 cm, 104.33 cm, 104.30 cm, 92.23 cm, 100.63 cm and 88.44 cm, respectively. Coefficients of variation for the 6 body measurement traits were 9.6, 8.0, 7.7, 7.9, 7.8 and 7.8, respectively.

**TABLE 1:** Descriptive statistics of 6 body measurement traits in Chinese Wenshan cattle.

Traits	Mean	SD	MAX	MIN	C.V%
BL	101.58	9.725	128.00	69.00	9.6
BH	104.33	8.375	142.00	63.00	8.0
WH	104.30	7.979	133.00	62.00	7.7
ITH	92.23	7.289	156.00	56.00	7.9
BAH	100.63	7.799	132.00	60.00	7.8
HH	88.44	6.868	117.00	54.00	7.8

SD, Standard Deviation; C.V, Coefficient of Variation.

The phenotypic correlation coefficients for the 6 body measurement traits were shown in Table 2. The results revealed that significant positive correlations were found between all of these 6 traits (*p* < 0.01), ranging from 0.603 to 0.961. The highest correlation was found between BH and BAH (r = 0.961, *p* < 0.001).

**TABLE 2:** Correlation coefficients of 6 body measurement traits in Chinese Wenshan cattle.

Traits (cm)	BL	BH	WH	ITH	BAH	HH
BL						
BH	0.678^**^					
WH	0.676^**^	0.920^**^				
ITH	0.603^**^	0.791^**^	0.829^**^			
BAH	0.670^**^	0.961^**^	0.935^**^	0.804^**^		
HH	0.629^**^	0.802^**^	0.841^**^	0.922^**^	0.818^**^	

**p < 0.01.

### 3.2 Population structure

**FIGURE 1:**
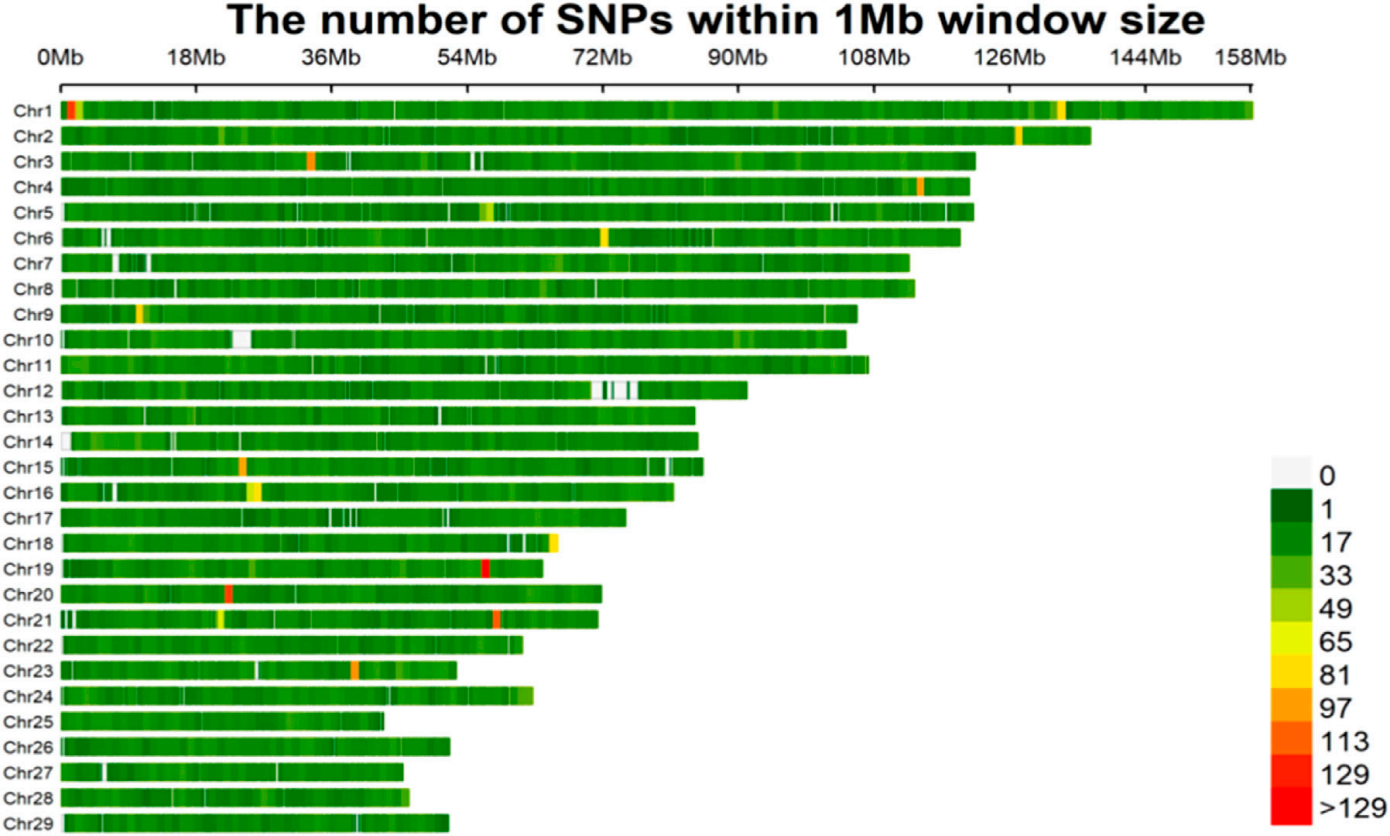
The filtered SNPs density distributions on chromosomes. The horizontal axis (*x*-axis) shows the chromosome length (Mb). Color index indicates the number of labels.

After elimination of the monomorphic loci and loci with minor allele frequency (MAF) below 1%, from the total of 63,791 SNP markers, 46,284 SNPs remained for associations with body measurement of Wenshan cattle. The density distributions of the filtered SNPs are shown in Figure 1. The highest number of markers was on chromosome 1 (3,168 SNPs), and the lowest number of markers was on chromosome 28 (824 SNPs). SNPs were found in almost all of the non-overlapping 1 Mb regions of the genome, indicating the reliability of the data.

**FIGURE 2:**
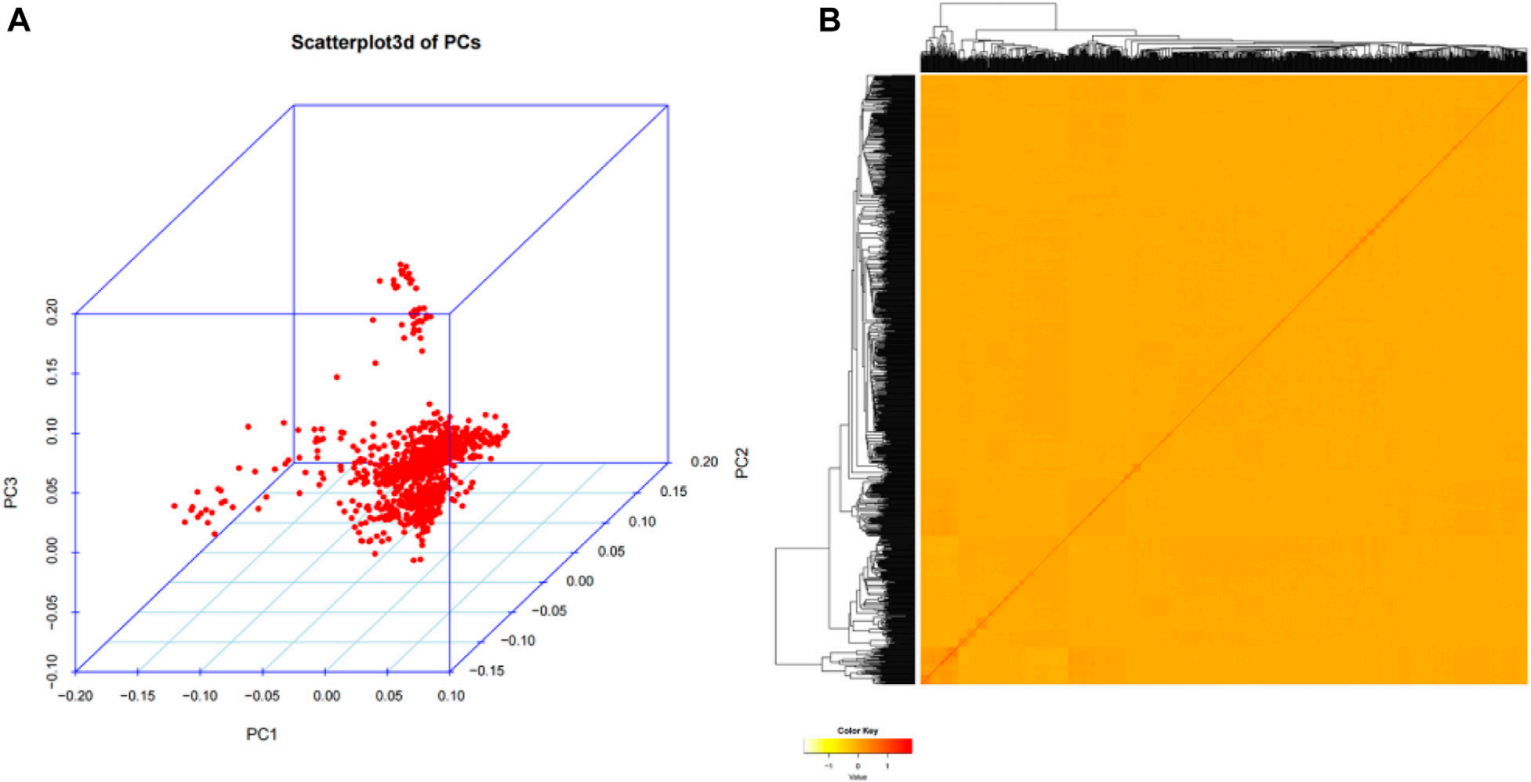
Population structure analysis. **(A)** The principal component analysis (PCA) for the third principal component (PC3) against the first principal component (PC1) and the second principal component (PC2). **(B)** The hierarchical clustering and heat map of the pairwise kinship matrix values.

In our study, in order to investigate the genetic structure of 1,060 Wenshan cattle, quality-controlled SNP markets were utilized for principal component analysis (PCA). The analysis revealed that there were no distinct genetic clusters among the samples, as shown in Figure 2A. Furthermore, the heatmap and dendrogram of the kinship matrix confirmed the absence of clear clusters in the population, indicating that the Wenshan cattle population in present study is irrelevant to family (Figure 2B).

### 3.3 GWAS of body measurement related traits

In total, 8 SNPs (listed in Table 3) were identified with the *p*-value ranging from 1.48 × 10^−5^ (Affx-277,062,550) to 2.3 × 10^−68^ (Affx-41,315,554), and the MAF ranging from 0.012 (Affx-113,744,044 and Affx-106,521,055) to 0.401 (Affx-277,062,550).

**TABLE 3:** Significant SNPs identified for 6 body measurement traits in Chinese Wenshan cattle.

SNP*	Chr	Position (bp)	MAF	*p-*value						SNP Effects					
				BH	BL	HH	WH	ITH	BAH	BH	BL	HH	WH	ITH	BAH
Affx-106521055	2	87,133,202	0.012		2.07E-05	5.57E-06	8.09E-06		1.27E-05		−9.72	−8.51	−9.65		-9.38
Affx-277062550	3	104,290,872	0.401	8.56E-06		4.18E-05	1.04E-05		7.91E-06	−2.55		−1.91	−2.21		−2.28
Affx-257095832	13	5,348,767	0.071	1.53E-64	3.05E-45	5.89E-63	3.48E-65	3.17E-62	6.79E-65	17.85	15.06	14.34	16.83	14.92	16.68
Affx-113744044	16	9,148,784	0.012	2.33E-05						11.13					
Affx-115873673	17	50,965,063	0.032	5.05E-23	6.84E-19	4.22E-23	2.25E-24	1.02E-26	7.03E-24	15.68	14.13	12.79	15.18	14.38	14.96
Affx-41315554	17	52,029,736	0.066	1.06E-67	1.58E-45	2.52E-64	6.26E-68	8.96E-64	2.30E-68	18.90	15.64	15.01	17.77	15.63	17.70
Affx-43208349	22	39,829,835	0.075	1.23E-61	1.61E-41	7.40E-57	7.53E-61	1.65E-56	1.51E-61	16.82	13.92	13.18	15.70	13.74	15.68
Affx-106517222	24	11,204,777	0.081		2.00E-05						−4.39				

For BH trait, a total of 6 SNPs were detected, with two located on chromosome 17 and the others on chromosomes 3, 13, 16 and 22, respectively. Among them, the most significant SNP was Affx-257,095,832 (*p* = 1.53 × 10^−64^), which located on BTA13: 5,348,767 bp. For BL trait, a total of 6 SNPs were detected, with two located on chromosome 17 and the others on chromosomes 2, 13, 22 and 24, respectively. Among them, the most significant SNP was Affx-41,315,554 (*p* = 1.58 × 10^−45^), which located on BTA17: 52,029,736 bp. For HH, WH and BAH trait, 6 identical SNPs were detected for every one of them, with two located on chromosome 17 and the others on chromosomes 2, 3, 13 and 22, respectively. For HH, WH and BAH, Affx-41,315,554 was the most significant one among their selected SNPs, which located on BTA17: 52,029,736 bp, and the *p*-values were 2.52 × 10^−64^, 6.26E×10^−68^ and 2.3 × 10^−68^, respectively. For ITH trait, a total of 4 SNPs were detected, with two located on chromosome 17 and the others on chromosomes 13 and 22, respectively. Among them, the most significant SNP was Affx-41,315,554 (*p* = 8.96 × 10^−64^), which located on BTA17: 52,029,736 bp.

**FIGURE 3:**
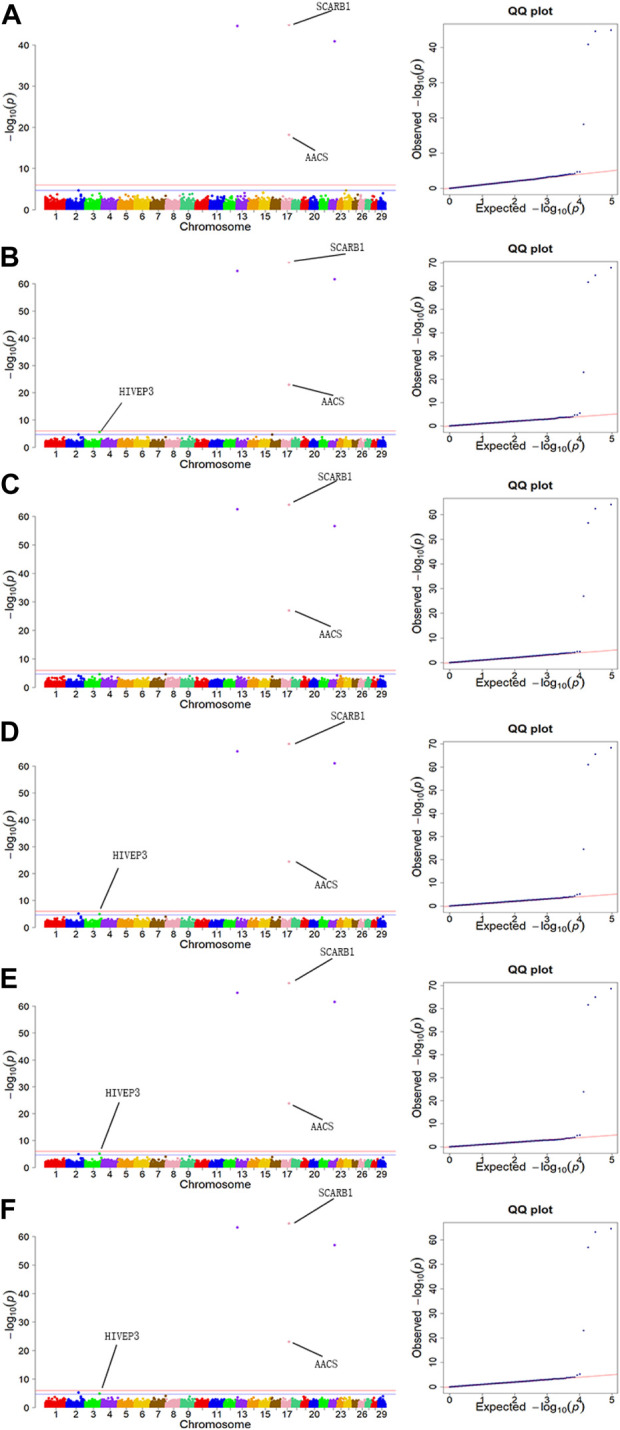
Manhattan plots and QQ plots for six body measurement traits using MLM. **(A)** BL, **(B)** BH, **(C)** ITH, **(D)** WH, **(E)** BAH, **(F)** HH. Negative log10 *p*-values of the filtered high-quality SNPs were plotted against their genomic positions. The solid lines of orange and blue correspond to the Bonferroni-corrected thresholds of *p* = 2.16 × 10^−5^ and *p* = 1.08 × 10^−6^, respectively.

The GWAS results for the 6 body measurement traits were illustrated by Manhattan plots and Quantile-Quantile (Q-Q) plots in Figure 3. QQ plots of BH, BL, WH, BAH, ITH and HH traits showed no evidence of population stratification in our study.

### 3.4 Common significant loci shared by three traits

**FIGURE 4:**
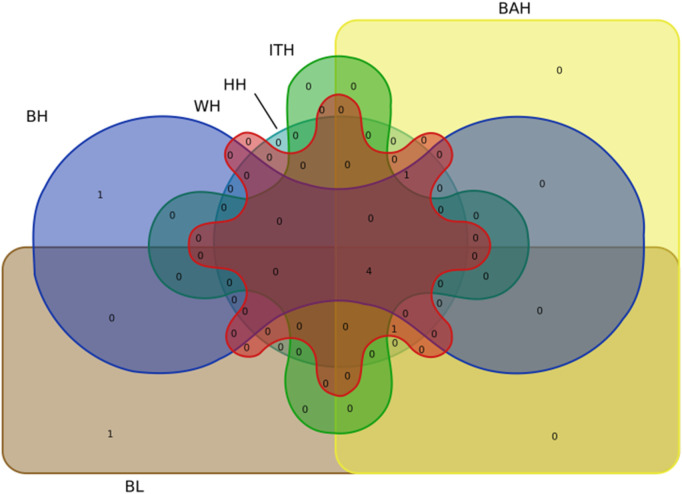
Venn diagram representation of the common significant loci in 6 body measurement traits for Chinese Wenshan cattle.

Particularly, we found that 4 significant SNPs including BTA13:5348767bp, BTA17:50965063bp, BTA17:52029736bp and BTA22:39829835bp were associated with all the 6 body measurement traits (Table 3; Figure 4). BTA3:104290872bp was associated with BH, WH, BAH and HH, respectively. BTA2:87133202bp was associated with BL, WH, BAH and HH, respectively.

### 3.5 Identification of candidate genes

To identify candidate genes associated with the 6 body measurement traits of Wenshan cattle, we searched for genes within a 0.5 Mb of the identified SNPs using the UCSC genome browser. In total, 21 genes were identified within or overlapping the candidate regions of 8 SNPs (Table 4). And, 1, 6, 13 and 1 genes were mapped for Affx-257,095,832, Affx-115,873,673, Affx-41,315,554 and Affx-277,062,550, respectively. Affx-106,521,055, Affx-113,744,044, Affx-43,208,349 and Affx-106,517,222 did not map any gene. Among these genes, 20 genes were associated with all the 6 traits, and mainly distributed on BTA13, 17 and 22. *HIVEP3*, which located on BTA3, was associated with WH, BAH, BH and HH, respectively. Through reviewing relevant research reports, we found that *Scarb1*, *AACS* and *HIVEP3* as candidate genes for body measurement traits according to their biological functions.

**TABLE 4:** List of candidate genes associated with 6 body measurement traits in Chinese Wenshan cattle.

Gene	Chr	Position (bp)	Related SNPs	Associated traits
*HIVEP3*	3	104,538,208–104,671,493	Affx-277,062,550	BH, HH, WH, BAH
*BTBD3*	13	5,455,770–5,474,367	Affx-257,095,832	BH, BL, HH, WH, BAH, ITH
*AACS*	17	50,718,350–50,773,650	Affx-115,873,673	
*BRI3BP*		50,813,518–5,0834,720		
*DHX37*		50,838,143–50,868,907		
*SNORA71*		50,869,224–50,869,321		
*UBC*		50,887,544–50,892,468		
*SCARB1*		50,929,375–51,022,457		
*CCDC92*		51,757,127–51,793,408		
*DNAH10*		51,794,311–51,952,067		
*ATP6V0A2*		51,954,039–51,993,763		
*TCTN2*		51,998,677–52,027,811	Affx-41,315,554	
*GTF2H3*		52,029,878–52,050,145		
*EIF2B1*		52,050,315–52,061,227		
*DDX55*		52,061,347–52,079,100		
*RILPL1*		52,111,245–52,153,382		
*SNRNP35*		52,157,896–52,161,635		
*RILPL2*		52,178,861–52,205,415		
*U6*		52,194,968–52,195,079		
*KMT5A*		52,211,310–52,228,130		
*SBNO1*		52,247,385–52,289,589		

## 4 Discussion

Population stratification is a crucial factor to consider in GWAS, as it can lead to an increased rate of false positives (Devlin and Roeder, 1999; Price et al., 2006; Kang et al., 2010; Zhou and Stephens, 2012; An et al., 2019). Many studies have indicated that adding population stratification to GWAS models can significantly improve the accuracy of analysis results (Devlin and Roeder, 1999; Price et al., 2006; Kang et al., 2010; An et al., 2019). MLM with principal component analysis (PCA) model was widely used to simulate population structure, kinship, and family structure, which is currently the most effective method to reduce population stratification (Price et al., 2006; Zhang et al., 2010). In the study of a commercial broiler chicken population, Mebratie, Wossenie et al. (2019) conducted GWAS using MLM approach, and found that using models that account for the population structure may reduce bias and increase accuracy of the estimated SNP effects in the association analysis. From the QQ plots (Figure 3), it can be seen that using the MLM model in our study can effectively reduce the impact of population stratification. Meanwhile, one of the major challenges in GWAS is multiple hypothesis testing (Joo et al., 2016). GWAS involves the assessment of millions of statistical tests, and as such, the *p*-value threshold for statistical significance must be adjusted to control the overall false positive rate. In order to control false positives or ensure the accuracy of analysis results, the Bonferroni correction approach which may be a little strict, and is often applied in GWAS (Al-Mamun et al., 2015; Puglisi et al., 2016; Abdalla et al., 2021). In the study for body measurement traits in Chinese Holstein cattle based on GWAS, 11 significant SNPs were identified using the Bonferroni correction approach, and identified 6 related genes (Abdalla et al., 2021). Thus, the Bonferroni was used as multiple hypothesis testing in this study and mapped 8 significant SNPs.

Body measurement traits in cattle are not only indicators of their physical development and size but are also important for assessing breed quality and production performance, such as weight. We observed a strong positive correlation among various body measurement traits, which is consistent with findings from other related studies. GUO et al. (2023) recently found Jinnan cattle exhibit a high positive correlation in body measurement traits (0.47–0.96), with a correlation of about 0.93 between WH and BH, which was aligns with our study (0.92). A study conducted by Afolayan et al. (2007) found that correlations between body measurements including HH and BL, at 400 days of age in Australian, and reported the values for phenotypic correlations were also highly positive (0.56–0.78). Another study (Kamprasert et al., 2019) found that the correlation between HH and BL of Brahman cattle at 400 days (0.80) was higher than our correlation of 0.629, but fell to 0.64 at 600 days, which indicated that the correlation between body measurement traits was influenced by both genetics and environment. Considering the strong correlation between multi body measurement traits, their genetic regulatory mechanisms should also have some similarities. In this study, we identified 8 significant SNPs, of which 6 appeared in more than one of the 6 body measurement traits, which was uniform with their phenotype correlations.

More importantly, sample size is the most critical factor that affects statistical power and limited sample size is hence a major hurdle in GWAS for traits that are difficult or expensive to measure (Gebreyesus et al., 2019). In this study, 1,060 Wenshan cattle were used for the GWAS of body measurement traits, with a much larger sample size than other similar studies in beef cattle (Buzanskas et al., 2014; An J. et al., 2019; Zepeda-Batista et al., 2021), thus ensuring the accuracy of our results. In this study, we identified three important genes including *Scarb1*, *AACS* and *HIVEP3*, which have been indicated to be related to bone metabolism, but there have been no previous studies on their association with livestock body measurement traits. These results indicate that those three genes may be specific to body measurement traits in Chinese Wenshan cattle and have high research value.

Scavenger receptor type I (*Scarb1*) on BTA17 was related to all the 6 body measurement traits. *Scarb1* produced by the *SCARB1* gene, is the major receptor for high-density lipoprotein (HDL). The role of *Scarb1* in bone formation has been the subject of conflicting findings in recent research. Martineau et al.(2014a; 2014b; 2014c) reported that Scarb1 knockout (KO) mice have increased trabecular bone and with no changes in osteoclasts parameters. In contrast, Tourkova et al. (2019) showed that *Scarb1* KO mice was osteopenic relative to the wild type. Morever, osteoblast and osteoclast-related mRNAs of KO mice greatly decreased compared to WT mice, suggesting that *Scarb1* is required for normal bone differentiation. More strangely, the latest research from Palmieri M et al. (2023a; 2023b) reported that bone mass was not affected in *Scarb1* KO mice and thought *Scarb1* did not contribute to bone homeostasis. However, it is established that HDL, via its main protein component apolipoprotein A1, is essential for normal bone formation by affecting osteoblast (Blair et al., 2016; Papachristou et al., 2017). Then, as the major receptor for HDL, we speculate that *Scarb1* may play an important role in bone formation, and relevant molecular mechanism needs further research.

*HIVEP3*, also known as Schnurri-3 (*SHN3*), is a big zinc finger protein and an essential regulator of bone formation. Jones C et al. (2006) found that *HIVEP3* ^−/−^ mice reveled osteosclerosis due to increased osteoblast activity and increased bone mass. Further research found that, *HIVEP3* regulated the bone mass by controlling the protein levels of *Runx2* which was the principal transcriptional regulator of osteoblast differentiation (Otto et al., 1997; Jones et al., 2006; Jones et al., 2007). Further investigation into the role of *HIVEP3* in osteoblasts has revealed that *HIVEP3* functions as a dampener of ERK (extracellular signal-regulated kinase) activity, particularly downstream of WNT signaling in osteoblasts. Mutations in the *HIVEP3* gene can disrupt this interaction, leading to abnormal activation of ERK and hyperactivity of osteoblasts *in vivo* (Shim et al., 2013). A genome-wide association analysis in Chinese populations suggested that the *HIVEP3* gene might be potentially correlated with Femoral Neck Bone Mineral Content (BMC) and Hip Geometry (Hu et al., 2020). By searching related researches, this study was the first to find that the *HIVEP3* gene had a potential relationship with BH, HH, WH, and BAH in the livestock population, especially in beef cattle. Thus, *HIVEP3* can be regarded as a candidate gene for Wenshan cattle body measurement traits.

The acetoacetyl-CoA synthetase (*AACS*) gene is known for the synthesis of biologically important lipogenic substances (Endemann et al., 1982). Previous reports have suggested that high level of serum lipids can trigger bone metabolic disorders (Bredella et al., 2013; Alsahli et al., 2016). In 1999, Mundy et al. (1949) found that statins are powerful cholesterol lowering medications that can improve new bone formation in rodents. Another study (Hasegawa et al., 2012) demonstrated that knockdown of *AACS in vivo* decreases peroxisome proliferator-activated receptor γ (PPARγ) and CCAAT/enhancer binding protein α (C/EBP α), which can enhance bone resorption and have a critical role in relationship between obesity and bone loss (Motyl et al., 2011; Akune et al., 2004). In obesity, *AACS* was highly expressed in the differentiated osteoclasts, but did not in osteoblast differentiation (Yamasaki et al., 2016). *In situ* hybridization, *AACS* was observed in several regions of the embryo, including the backbone region (especially the somite) and adult femur epiphysis. Collectively, these findings indicated that *AACS* may be involved in bone homeostasis through its impact on adipogenic transcription factors such as the C/EBP family and PPARγ. Then, we speculate that *AACS* plays an important role associated with the bone homeostasis, which is worthy of further study and can be selected as a candidate gene for body measurement traits.

## 5 Conclusion

To sum up, 8 SNPs were detected to be associated with 6 body measurement traits. Interestingly, 6 of these SNPs were detected in more than one of our 6 body measurement traits, which was consistent with their phenotype correlations. Meanwhile, we found 21 candidate genes located nearby or within the associated SNPs. Among them, *Scarb1, AACS* and *HIVEP3* have been indicated to be related to bone formation and are also discovered rarely in livestock body measurement traits, which can be used as candidate genes for Wenshan cattle. This study offers valuable insights for the further investigation of potential genetic mechanism of body measurement traits in Wenshan cattle.

## Data Availability

The datasets presented in this study can be found in online repositories. The names of the repository/repositories and accession number(s) can be found below: https://bigd.big.ac.cn/gsa/browse/CRA012611, PRJCA019725.

## References

[B1] Abdalla I. M. Lu X. Nazar M. Arbab A. A. I. Xu T. Yousif M. H. (2021). Genome-wide association study identifies candidate genes associated with feet and leg conformation traits in Chinese Holstein cattle. Anim. (Basel) 11, 2259. 10.3390/ani11082259 PMC8388412 34438715

[B2] Afolayan R. A. Pitchford W. S. Deland M. P. McKiernan W. A. (2007). Breed variation and genetic parameters for growth and body development in diverse beef cattle genotypes. Animal 1, 13–20. 10.1017/S1751731107257933 22444205

[B45] Akune T. Ohba S. Kamekura S. Yamaguchi M. Chung U. I. Kubota N. (2004). PPARgamma insufficiency enhances osteogenesis through osteoblast formation from bone marrow progenitors. The Journal of clinical investigation 113, 846–855. 10.1172/JCI19900 15067317 PMC362117

[B3] Al-Mamun H. A. Kwan P. Clark S. A. Ferdosi M. H. Tellam R. Gondro C. (2015). Genome-wide association study of body weight in Australian Merino sheep reveals an orthologous region on OAR6 to human and bovine genomic regions affecting height and weight. Genet. Sel. Evol. 47, 66. 10.1186/s12711-015-0142-4 26272623 PMC4536601

[B4] Alsahli A. Kiefhaber K. Gold T. Muluke M. Jiang H. Cremers S. (2016). Palmitic acid reduces circulating bone formation markers in obese animals and impairs osteoblast activity via C16-ceramide accumulation. Calcif. Tissue Int. 98, 511–519. 10.1007/s00223-015-0097-z 26758875

[B5] An B. Xia J. Chang T. Wang X. Xu L. Zhang L. (2019a). Genome-wide association study reveals candidate genes associated with body measurement traits in Chinese Wagyu beef cattle. Anim. Genet. 50, 386–390. 10.1111/age.12805 31179577

[B6] An B. Xu L. Xia J. Wang X. Miao J. Chang T. (2020). Multiple association analysis of loci and candidate genes that regulate body size at three growth stages in Simmental beef cattle. BMC Genet. 21, 32. 10.1186/s12863-020-0837-6 32171250 PMC7071762

[B7] An J. Won S. Lutz S. M. Hecker J. Lange C. (2019b). Effect of population stratification on SNP-by-environment interaction. Genet. Epidemiol. 43, 1046–1055. 10.1002/gepi.22250 31429121 PMC6829023

[B8] Benfica L. F. Sakamoto L. S. Magalhães A. F. B. de Oliveira M. H. V. de Albuquerque L. G. Cavalheiro R. (2020). Genetic association among feeding behavior, feed efficiency, and growth traits in growing indicine cattle. J. Anim. Sci. 98, skaa350. 10.1093/jas/skaa350 33125460 PMC7751144

[B9] Bessa A. F. O. Duarte I. N. H. Rola L. D. Bernardes P. A. Gonzaga Neto S. Lôbo R. B. (2021). Genetic evaluation for reproductive and productive traits in Brahman cattle. Theriogenology 173, 261–268. 10.1016/j.theriogenology.2021.08.008 34403971

[B10] Blair H. C. Kalyvioti E. Papachristou N. I. Tourkova I. L. Syggelos S. A. Deligianni D. (2016). Apolipoprotein A-1 regulates osteoblast and lipoblast precursor cells in mice. Lab. Invest. 96, 763–772. 10.1038/labinvest.2016.51 27088511

[B11] Bouwman A. C. Daetwyler H. D. Chamberlain A. J. Ponce C. H. Sargolzaei M. Schenkel F. S. (2018). Meta-analysis of genome-wide association studies for cattle stature identifies common genes that regulate body size in mammals. Nat. Genet. 50, 362–367. 10.1038/s41588-018-0056-5 29459679

[B12] Bredella M. A. Gill C. M. Gerweck A. V. Landa M. G. Kumar V. Daley S. M. (2013). Ectopic and serum lipid levels are positively associated with bone marrow fat in obesity. Radiology 269, 534–541. 10.1148/radiol.13130375 23861502 PMC3807082

[B13] Browning B. L. Browning S. R. (2009). A unified approach to genotype imputation and haplotype-phase inference for large data sets of trios and unrelated individuals. Am. J. Hum. Genet. 84, 210–223. 10.1016/j.ajhg.2009.01.005 19200528 PMC2668004

[B14] Buzanskas M. E. Grossi D. A. Ventura R. V. Schenkel F. S. Sargolzaei M. Meirelles S. L. (2014). Genome-wide association for growth traits in Canchim beef cattle. PLoS One 9, e94802. 10.1371/journal.pone.0094802 24733441 PMC3986245

[B15] Chen Q. Zhan J. Shen J. Qu K. Hanif Q. Liu J. (2020). Whole-genome resequencing reveals diversity, global and local ancestry proportions in Yunling cattle. J. Anim. Breed. Genet. 137, 641–650. 10.1111/jbg.12479 32297417

[B16] Chen Z. Chu S. Xu X. Jiang J. Wang W. Shen H. (2019). Analysis of longissimus muscle quality characteristics and associations with DNA methylation status in cattle. Genes Genomics 41, 1147–1163. 10.1007/s13258-019-00844-4 31256337

[B17] China National Commission of Animal Genetic Resources (2011). Animal genetic resources in China bovines. Beijing: Chinese Agricultural Press, 175–178. (In Chinese).

[B18] Dawson C. R. Henley P. A. Schroeder A. R. Meteer W. T. Hayes C. A. Felix T. L. (2022). Effects of rubber matting on feedlot cattle growth performance, locomotion, and carcass characteristics in slatted floor facilities. J. Anim. Sci. 100, skac041. 10.1093/jas/skac041 35148402 PMC9030117

[B19] Devlin B. Roeder K. (1999). Genomic control for association studies. Biometrics 55, 997–1004. 10.1111/j.0006-341x.1999.00997.x 11315092

[B20] Endemann G. Goetz P. G. Edmond J. Brunengraber H. (1982). Lipogenesis from ketone bodies in the isolated perfused rat liver. Evidence for the cytosolic activation of acetoacetate. J. Biol. Chem. 257, 3434–3440. 10.1016/s0021-9258(18)34796-3 7061490

[B21] Fan Y. Han Z. Arbab A. A. I. Yang Y. Yang Z. (2020). Effect of aging time on meat quality of longissimus dorsi from Yunling cattle: a new hybrid beef cattle. Animals 10, 1897. 10.3390/ani10101897 33081174 PMC7602736

[B22] Forabosco F. Groen A. F. Bozzi R. Van Arendonk J. A. Filippini F. Boettcher P. (2004). Phenotypic relationships between longevity, type traits, and production in Chianina beef cattle. J. Anim. Sci. 82, 1572–1580. 10.2527/2004.8261572x 15216982

[B23] Gebreyesus G. Buitenhuis A. J. Poulsen N. A. Visker M. H. P. W. Zhang Q. van Valenberg H. J. F. (2019). Combining multi-population datasets for joint genome-wide association and meta-analyses: the case of bovine milk fat composition traits. J. Dairy Sci. 102, 11124–11141. 10.3168/jds.2019-16676 31563305

[B24] Gritsenko S. Ruchay A. Kolpakov V. Lebedev S. Guo H. Pezzuolo A. (2023). On-barn forecasting beef cattle production based on automated non-contact body measurement system. Anim. (Basel) 13, 611. 10.3390/ani13040611 PMC9951648 36830398

[B25] Guo M. P. Meng Y. Wang H. H. Wang Y. Che L. J. Shen L. (2023). Estimation of genetic parameters for body weight and size traits in Jinnan cattle at different growth stages. Acta Veterinaria Zootech. Sin. 54, 1452–1464. (in Chinese). 10.11843/j.issn.0366-6964.2023.04.010

[B26] Hasegawa S. Ikeda Y. Yamasaki M. Fukui T. (2012). The role of acetoacetyl-CoA synthetase, a ketone body-utilizing enzyme, in 3T3-L1 adipocyte differentiation. Biol. Pharm. Bull. 35, 1980–1985. 10.1248/bpb.b12-00435 23123469

[B27] Hu W. He J. Qi L. Wang C. Yue H. Gu J. (2020). Association of HIVEP3 gene and lnc RNA with femoral Neck bone mineral content and hip geometry by genome-wide association analysis in Chinese people. Int. J. Endocrinol. 2020, 6929073. 10.1155/2020/6929073 33110425 PMC7579678

[B28] Jones D. C. Wein M. N. Glimcher L. H. (2007). Schnurri-3 is an essential regulator of osteoblast function and adult bone mass. Ann. Rheum. Dis. 66, iii49–iii51. 10.1136/ard.2007.078352 17934096 PMC2095286

[B29] Jones D. C. Wein M. N. Oukka M. Hofstaetter J. G. Glimcher M. J. Glimcher L. H. (2006). Regulation of adult bone mass by the zinc finger adapter protein Schnurri-3. Science 312, 1223–1227. 10.1126/science.1126313 16728642

[B30] Joo J. W. Hormozdiari F. Han B. Eskin E. (2016). Multiple testing correction in linear mixed models. Genome Biol. 17, 62. 10.1186/s13059-016-0903-6 27039378 PMC4818520

[B31] Kamprasert N. Duijvesteijn N. Van der Werf J. H. J. (2019). Estimation of genetic parameters for BW and body measurements in Brahman cattle. Animal 13, 1576–1582. 10.1017/S1751731118003348 30614434

[B32] Kang H. M. Sul J. H. Service S. K. Zaitlen N. A. Kong S. Y. Freimer N. B. (2010). Variance component model to account for sample structure in genome-wide association studies. Nat. Genet. 42, 348–354. 10.1038/ng.548 20208533 PMC3092069

[B33] Karimi K. Esmailizadeh Koshkoiyeh A. Gondro C. (2015). Comparison of linkage disequilibrium levels in Iranian indigenous cattle using whole genome SNPs data. J. Anim. Sci. Technol. 57, 47. 10.1186/s40781-015-0080-2 26705480 PMC4690407

[B34] Larson H. E. Jaderborg J. P. Paulus-Compart D. M. Crawford G. I. DiCostanzo A. (2023). Effect of substitution of distillers grains and glycerin for steam-flaked corn in finishing cattle diets on growth performance and carcass characteristics. J. Anim. Sci. 101, skac348. 10.1093/jas/skac348 36592746 PMC9831090

[B35] Li M. Lu X. Xia H. Zhang C. Wang X. Chen Z. (2019). In-depth characterization of the pituitary transcriptome in Simmental and Chinese native cattle. Domest. Anim. Endocrinol. 66, 35–42. 10.1016/j.domaniend.2018.09.003 30391830

[B36] Li Y. Samartzis D. Campbell D. D. Cherny S. S. Cheung K. M. Luk K. D. (2016). Two subtypes of intervertebral disc degeneration distinguished by large-scale population-based study. Spine J. 16, 1079–1089. 10.1016/j.spinee.2016.04.020 27157501

[B37] Liu Y. Cheng H. Wang S. Luo X. Ma X. Sun L. (2022). Genomic diversity and selection signatures for Weining Cattle on the border of Yunnan-Guizhou. Front. Genet. 13, 848951. 10.3389/fgene.2022.848951 35873486 PMC9301131

[B38] MacDonald J. M. (2003). Beef and pork packing industries. Vet. Clin. North Am. Food Anim. Pract. 19, 419–443. 10.1016/s0749-0720(03)00022-7 12951741

[B39] Martineau C. Kevorkova O. Brissette L. Moreau R. (2014c). Scavenger receptor class B, type I (Scarb1) deficiency promotes osteoblastogenesis but stunts terminal osteocyte differentiation. Physiol. Rep. 2, e12117. 10.14814/phy2.12117 25281615 PMC4254088

[B40] Martineau C. Martin-Falstrault L. Brissette L. Moreau R. (2014a). The atherogenic Scarb1 null mouse model shows a high bone mass phenotype. Am. J. Physiol. Endocrinol. Metab. 306, E48–E57. 10.1152/ajpendo.00421.2013 24253048 PMC3920004

[B41] Martineau C. Martin-Falstrault L. Brissette L. Moreau R. (2014b). Gender- and region-specific alterations in bone metabolism in Scarb1-null female mice. J. Endocrinol. 222, 277–288. 10.1530/JOE-14-0147 24928939

[B42] McKay S. D. Schnabel R. D. Murdoch B. M. Matukumalli L. K. Aerts J. Coppieters W. (2007). Whole genome linkage disequilibrium maps in cattle. BMC Genet. 8, 74. 10.1186/1471-2156-8-74 17961247 PMC2174945

[B43] Mebratie W. Reyer H. Wimmers K. Bovenhuis H. Jensen J. (2019). Genome wide association study of body weight and feed efficiency traits in a commercial broiler chicken population, a re-visitation. Sci. Rep. 9, 922. 10.1038/s41598-018-37216-z 30696883 PMC6351590

[B44] Meng X. Gao Z. Liang Y. Zhang C. Chen Z. Mao Y. (2020). Longissimus dorsi muscle transcriptomic analysis of simmental and Chinese native cattle differing in meat quality. Front. Vet. Sci. 7, 601064. 10.3389/fvets.2020.601064 33385016 PMC7770222

[B46] Motyl K. J. Raetz M. Tekalur S. A. Schwartz R. C. McCabe L. R. (2011). CCAAT/enhancer binding protein β-deficiency enhances type 1 diabetic bone phenotype by increasing marrow adiposity and bone resorption. Am. J. Physiol. Regul. Integr. Comp. Physiol. 300, R1250–R1260. 10.1152/ajpregu.00764.2010 21346244 PMC3094035

[B47] Mundy G. Garrett R. Harris S. Chan J. Chen D. Rossini G. (1949). Stimulation of bone formation *in vitro* and in rodents by statins. Science 286, 1946–1949. 10.1126/science.286.5446.1946 10583956

[B48] Munim T. Oikawa T. Ibi T. Kunieda T. (2013). Genetic relationship of body measurement traits at early age with carcass traits in Japanese black cattle. Anim. Sci. J. 84, 206–212. 10.1111/asj.12005 23480700

[B49] Naserkheil M. Lee D. H. Mehrban H. (2020). Improving the accuracy of genomic evaluation for linear body measurement traits using single-step genomic best linear unbiased prediction in Hanwoo beef cattle. BMC Genet. 21, 144. 10.1186/s12863-020-00928-1 33267771 PMC7709290

[B50] Nie L. Yu Y. Zhang X. Q. Yang G. F. Wen J. K. Zhang Y. P. (1999). Genetic diversity of cattle in south China as revealed by blood protein electrophoresis. Biochem. Genet. 37, 257–265. 10.1023/a:1018798924778 10624516

[B51] Niu Q. Zhang T. Xu L. Wang T. Wang Z. Zhu B. (2021). Integration of selection signatures and multi-trait GWAS reveals polygenic genetic architecture of carcass traits in beef cattle. Genomics 113, 3325–3336. 10.1016/j.ygeno.2021.07.025 34314829

[B52] Otto F. Thornell A. P. Crompton T. Denzel A. Gilmour K. C. Rosewell I. R. (1997). Cbfa1, a candidate gene for cleidocranial dysplasia syndrome, is essential for osteoblast differentiation and bone development. Cell 89, 765–771. 10.1016/s0092-8674(00)80259-7 9182764

[B53] Palmieri M. Joseph T. E. O'Brien C. A. Gomez-Acevedo H. Kim H. N. Manolagas S. C. (2023b). Retracted: deletion of the scavenger receptor Scarb1 in myeloid cells does not affect bone mass. Bone 170, 116702. 10.1016/j.bone.2023.116702 36773885 PMC10040251

[B54] Palmieri M. Joseph T. E. O'Brien C. A. Gomez-Acevedo H. Manolagas S. C. Ambrogini E. (2023a). Retraction: deletion of the scavenger receptor Scarb1 in osteoblast progenitors does not affect bone mass. PLoS One 18, e0290458. 10.1371/journal.pone.0290458 37585400 PMC10431598

[B55] Papachristou N. I. Blair H. C. Kypreos K. E. Papachristou D. J. (2017). High-density lipoprotein (HDL) metabolism and bone mass. J. Endocrinol. 233, R95-R107–R107. 10.1530/JOE-16-0657 28314771 PMC5598779

[B56] Porto-Neto L. R. Kijas J. W. Reverter A. (2014). The extent of linkage disequilibrium in beef cattle breeds using high-density SNP genotypes. Genet. Sel. Evol. 46, 22. 10.1186/1297-9686-46-22 24661366 PMC4021229

[B57] Price A. L. Patterson N. J. Plenge R. M. Weinblatt M. E. Shadick N. A. Reich D. (2006). Principal components analysis corrects for stratification in genome-wide association studies. Nat. Genet. 38, 904–909. 10.1038/ng1847 16862161

[B58] Puglisi R. Gaspa G. Balduzzi D. Severgnini A. Vanni R. Macciotta N. (2016). Genome-wide analysis of bull sperm quality and fertility traits. Reprod. Domest. Anim. 51, 840–843. 10.1111/rda.12747 27550832

[B59] Purcell S. Neale B. Todd-Brown K. Thomas L. Ferreira M. A. Bender D. (2007). PLINK: a tool set for whole-genome association and population-based linkage analyses. Am. J. Hum. Genet. 81, 559–575. 10.1086/519795 17701901 PMC1950838

[B60] Reich D. E. Cargill M. Bolk S. Ireland J. Sabeti P. C. Richter D. J. (2011). Linkage disequilibrium in the human genome. Nature 411, 199–204. 10.1038/35075590 11346797

[B61] Ring S. C. Graham D. A. Kelleher M. M. Doherty M. L. Berry D. P. (2019). Genetic parameters for variability in the birth of persistently infected cattle following likely *in utero* exposure to bovine viral diarrhea virus. J. Anim. Sci. 97, 559–568. 10.1093/jas/sky430 30412254 PMC6358239

[B62] Schmidtmann C. Segelke D. Bennewitz J. Tetens J. Thaller G. (2023). Genetic analysis of production traits and body size measurements and their relationships with metabolic diseases in German Holstein cattle. J. Dairy Sci. 106, 421–438. 10.3168/jds.2022-22363 36424319

[B63] Setati M. M. Norris D. Banga C. B. Benyi K. (2004). Relationships between longevity and linear type traits in Holstein cattle population of Southern Africa. Trop. Anim. Health Prod. 36, 807–814. 10.1023/b:trop.0000045965.99974.9c 15643816

[B64] Shim J. H. Greenblatt M. B. Zou W. Huang Z. Wein M. N. Brady N. (2013). Schnurri-3 regulates ERK downstream of WNT signaling in osteoblasts. J. Clin. Invest. 123, 4010–4022. 10.1172/JCI69443 23945236 PMC3754267

[B65] Shin S. Lee J. Do C. (2021). Genetic relationship of age at first calving with conformation traits and calving interval in Hanwoo cows. J. Anim. Sci. Technol. 63, 740–750. 10.5187/jast.2021.e73 34447951 PMC8367412

[B66] Singh A. Kumar A. Mehrotra A. Pandey A. K. Mishra B. P. (2021). Estimation of linkage disequilibrium levels and allele frequency distribution in crossbred Vrindavani cattle using 50K SNP data. PLoS One 16, e0259572. 10.1371/journal.pone.0259572 34762692 PMC8584695

[B67] Song H. Li W. Li Y. Zhai B. Guo Y. Chen Y. (2023). Genome-wide association study of 17 serum biochemical indicators in a chicken F2 resource population. BMC Genomics 24, 98. 10.1186/s12864-023-09206-7 36864386 PMC9983160

[B68] Steel R. Torrie J. H. (1997). “Analysis of variance II: multiway classifications,” in Principles and procedures of statistics: a biometrical approach (NY: McGraw-Hill), 204–252.

[B69] Terakado A. P. N. Costa R. B. de Camargo G. M. F. Irano N. Bresolin T. Takada L. (2018). Genome-wide association study for growth traits in Nelore cattle. Animal 12, 1358–1362. 10.1017/S1751731117003068 29143708

[B70] Tourkova I. L. Dobrowolski S. F. Secunda C. Zaidi M. Papadimitriou-Olivgeri I. Papachristou D. J. (2019). The high-density lipoprotein receptor Scarb1 is required for normal bone differentiation *in vivo* and *in vitro* . Lab. Invest. 99, 1850–1860. 10.1038/s41374-019-0311-0 31467425 PMC11715553

[B71] van den Berg I. Ho P. N. Nguyen T. V. Haile-Mariam M. MacLeod I. M. Beatson P. R. (2022). GWAS and genomic prediction of milk urea nitrogen in Australian and New Zealand dairy cattle. Genet. Sel. Evol. 54, 15. 10.1186/s12711-022-00707-9 35183113 PMC8858489

[B72] Vanvanhossou S. F. U. Scheper C. Dossa L. H. Yin T. Brügemann K. König S. (2020). A multi-breed GWAS for morphometric traits in four Beninese indigenous cattle breeds reveals loci associated with conformation, carcass and adaptive traits. BMC Genomics 21, 783. 10.1186/s12864-020-07170-0 33176675 PMC7656759

[B73] Wu G. (2020). Important roles of dietary taurine, creatine, carnosine, anserine and 4-hydroxyproline in human nutrition and health. Amino acids 52, 329–360. 10.1007/s00726-020-02823-6 32072297 PMC7088015

[B74] Yamasaki M. Hasegawa S. Imai M. Takahashi N. Fukui T. (2016). High-fat diet-induced obesity stimulates ketone body utilization in osteoclasts of the mouse bone. Biochem. Biophys. Res. Commun. 473, 654–661. 10.1016/j.bbrc.2016.03.115 27021680

[B75] Yan C. L. Lin J. Huang Y. Y. Gao Q. S. Piao Z. Y. Yuan S. L. (2022). Population genomics reveals that natural variation in PRDM16 contributes to cold tolerance in domestic cattle. Zool. Res. 43, 275–284. 10.24272/j.issn.2095-8137.2021.360 35238185 PMC8920848

[B76] Yin L. Zhang H. Tang Z. Xu J. Yin D. Zhang Z. (2021). rMVP: a memory-efficient, visualization-enhanced, and parallel-accelerated tool for genome-wide association study. Proteomics Bioinforma. 19 (4), 619–628. 10.1016/j.gpb.2020.10.007 PMC9040015 33662620

[B77] Zepeda-Batista J. L. Núñez-Domínguez R. Ramírez-Valverde R. Jahuey-Martínez F. J. Herrera-Ojeda J. B. Parra-Bracamonte G. M. (2021). Discovering of genomic variations associated to growth traits by GWAS in Braunvieh cattle. Genes 12, 1666. 10.3390/genes12111666 34828272 PMC8618990

[B78] Zhang Y. Zhang J. Gong H. Cui L. Zhang W. Ma J. (2019). Genetic correlation of fatty acid composition with growth, carcass, fat deposition and meat quality traits based on GWAS data in six pig populations. Meat Sci. 150, 47–55. 10.1016/j.meatsci.2018.12.008 30584983

[B79] Zhang Z. Ersoz E. Lai C. Q. Todhunter R. J. Tiwari H. K. Gore M. A. (2010). Mixed linear model approach adapted for genome-wide association studies. Nat. Genet. 42, 355–360. 10.1038/ng.546 20208535 PMC2931336

[B80] Zhou X. Stephens M. (2012). Genome-wide efficient mixed-model analysis for association studies. Nat. Genet. 44, 821–824. 10.1038/ng.2310 22706312 PMC3386377

